# Immune Complexes of Beta-2-Glycoprotein I and IgA Antiphospholipid Antibodies Identify Patients With Elevated Risk of Thrombosis and Early Mortality After Heart Transplantation

**DOI:** 10.3389/fimmu.2019.02891

**Published:** 2019-12-23

**Authors:** Manuel Serrano, Laura Morán, Jose Angel Martinez-Flores, Esther Mancebo, Daniel Pleguezuelo, Oscar Cabrera-Marante, Juan Delgado, Antonio Serrano

**Affiliations:** ^1^Immunology Department, Healthcare Research Institute, Hospital “12 de Octubre”, Madrid, Spain; ^2^Immunology Department, Hospital Clínico San Carlos, Madrid, Spain; ^3^Cardiology Department, CIBERCV, Facultad de Medicina, Healthcare Research Institute, Hospital “12 de Octubre”, Universidad Complutense de Madrid, Madrid, Spain; ^4^Biomedical Research Centre Network for Epidemiology and Public Health (CIBERESP), Madrid, Spain

**Keywords:** antiphospholipid, heart transplant, circulating immune-complexes, anti-beta-2-glycoprotein I, non-criteria aPL, IgA

## Abstract

**Background:** The presence of anti-Beta 2 glycoprotein antibodies (aB2GP1) of IgA isotype is common in patients with functional impairment of the organs in which B2GP1 is elaborated. Pretransplant IgA aB2GP1 has been associated with increased risk of thrombosis in kidney and heart transplanted patients and has also been related with early mortality after heart transplantation. Circulating immune complexes between IgA and B2GP1 (B2A-CIC) have been described in the blood of patients positive for IgA aB2GP1 with thrombotic clinical symptoms. In kidney transplanted patients, B2A-CIC is a biomarker that predicts which patients IgA aB2GP1 positive are at risk of thrombosis events following kidney transplantation and may lead to early prophylactic treatment. The prevalence of B2A-CIC and its relation with outcomes after heart transplantation is not known.

**Methods:** Follow-up study based on 151 consecutive patients who received a heart transplant. Autoantibodies and B2A-CIC were quantified in pre-transplant serum samples. Three groups of patients were followed-up for 2 years: Group-1, positive for IgA aB2GP1 and B2A-CIC (*N* = 19). Group-2, only positive for IgA aB2GP1 (*N* = 28). Group-0 (control group): IgA aB2GP1 negative (*N* = 104).

**Results:** Kaplan-Meir survival analysis showed that mortality in B2A-CIC positive was higher than group-0 at 3 months (HR:5.08; 95%CI: 1.36–19.01) and at 2 years (HR:3.82; 95%CI: 1.54–12.66). No significant differences were observed between group-2 and group-0. Multivariate analysis identified B2A-CIC as the most important independent risk factor for early mortality (OR = 6.12; 95% CI: 1.93–19.4). Post-transplant incidence of thrombosis was significantly higher in B2A-CIC positive patients than in the control group (OR: 6.42; 95%CI: 2.1–19.63). Multivariate analysis identified the presence of B2A-CIC (OR: 6.13; 95%CI: 2.1–19.63) and the pre-transplant habit of smoking actively (OR: 4.18; 95%CI: 1.35–12.94) as independent risk factor for thrombosis. The proportion of patients who had thrombotic events or died in the first trimester was significantly higher in group-1 (73.7%) than in group-0 (16.3%; *p* < 0.001) and in group-2 (39.3%; *p* = 0.02). Multivariate analysis identified B2A-CIC as the main independent risk factor for early outcomes (mortality or thrombosis) in the first 3 months after heart transplant (OR = 11.42, 95% CI: 1.69–9.68).

**Conclusion:** B2A-CIC are a predictor of early mortality and thrombosis after heart transplant.

## Key Messages

- Pre-transplant prevalence of B2A-CIC in heart transplanted patients is 12.6%.
- Pre-transplant presence of B2A-CIC is the main independent risk factor for mortality in the first 3 months after heart transplantation.
- About ¾ of B2A-CIC positive patients suffer thrombotic events or death in the first 3 months after heart transplant.
- Positivity of B2A-CIC identifies patients with higher risk of mortality and incidence of thrombotic events after heart transplantation.

## Introduction

Primary Antiphospholipid Syndrome (P-APS) is an acquired autoimmune disorder characterized by: (1) The presence of recurrent venous or arterial thrombosis and/or gestational morbidity. (2) The presence in the blood of antiphospholipid antibodies (APL). (3) The absence of other systemic autoimmune diseases (1–4).

The autoantibodies included in the classification criteria for antiphospholipid syndrome (APS) are the presence of lupus anticoagulant, or the presence of IgG or IgM isotype antibodies directed against cardiolipin (aCL) or against B2-Glycoprotein I (aB2GP1) (5, 6).

Several authors have suggested that the assessment of new autoantibodies can help to identify the syndrome in patients with APS clinic (7–10). Among the new aPL that have been described associated with APS-events, the IgA aB2GP1 and the anti-phosphatidyl serine/prothrombin (IgG or IgM) are those where a greater association with the APS clinical aspects has been observed (11–13). The clinical relevance of IgA aB2GPI has increased progressively in recent years. In the 13th International Congress on Antiphospholipid Antibodies (2010, Galveston, TX), the task force recommended testing for the IgA aB2GPI in patients with a clinical profile suggestive of APS and negative for the aPL that are included in the APS classification criteria (11).

Our group has described an elevated prevalence of IgA aB2GP1 in patients with chronic kidney disease (CKD) vs. the general population (30 vs. 1.5%) and the association between presence of these antibodies with thrombotic events and mortality in these patients (12). This observation has also been confirmed in other cohorts (13). Patients with CDK positive for IgA aB2GP1 who received a kidney transplant showed a greater incidence of early graft loss, mainly due to thrombosis (14, 15). This fact has been confirmed in a prospective multicenter study (16).

As the presence of these antibodies was not associated with genetic factors (17), type of renal function replacement treatment (12) or base disease that caused renal failure (15), we proposed the hypothesis that misfolded B2GP1 produced in the stressed cells of an unhealthy organ could be identified as a foreign antigen (14). This hypothesis could be supported by the works of Arase et al. that demonstrated that misfolded self-proteins can be incorporated into MHC class II without previous processing and can be transported in their entire form to the cell surface (18). The same group describes the presence of complexes B2GP1- HLA class II as autoantigens in antiphospholipid syndrome (19).

B2GP1 is a plasma protein that is elaborated mainly in the liver, but also in other organs such as the kidney and the heart (20). In this way, we studied the prevalence of IgA aB2GP1 in patients with severe heart failure waiting for heart transplantation, finding a prevalence similar to that observed in CKD as well as a strong association with mortality and thrombosis after heart transplantation (21).

Although the presence of IgA aB2GP1 identifies many patients at risk of developing thrombotic events after kidney transplantation, its predictive value is too low to justify applying preventive treatments to patients who express this biomarker. Searching for new biomarkers that would allow a better identification of the population at risk, circulating immune complexes (CIC) of IgA bound to B2GP1 (B2A-CIC) were described in the blood of patients with APS symptomatology (22) and its presence was associated with the occurrence of acute thrombotic events (23).

In patients who will undergo a kidney transplant, the pretransplant presence of B2A-CIC helps to identify which patients with antiphospholipid antibodies have high risk of developing thrombosis in the first 6 months after kidney transplantation. Patients positive for IgA aB2GP1 who were negative for B2A-CIC had a thrombosis risk similar to the general population (24).

In this work we will assess whether the presence of pre-transplant B2A-CIC allows to better identify the population with higher risk of developing mortality, cardiovascular events and thrombosis in the 24 first months after heart transplantation.

## Methods

### Study Design

We carried out a 2-year follow-up study using the historical cohort “H12 + 8” that included all patients (*N* = 153) who had received a heart transplant over a period of 8 years (01/01/2004 to 12/31/2011) in the ´Hospital 12 de Octubreˇ (Madrid, Spain) (21).

Aim: To determine the pretransplant prevalence of B2A-CIC in patients positive for IgA aB2GP1 and investigate their possible association with mortality, thrombosis and other cardiovascular events after the transplant.

Main endpoints: thrombosis, vascular events, death, patient survival at three and 24 months.

### Patients

A total of 151 consecutive patients who underwent heart transplantation in a period of 8 years in a single center were enrolled and studied for 24 months or until death. Two patients of the original cohort were excluded: one patient who had received two heart transplants was only included in the second transplant and a second patient lacked a pretransplant serum sample.

Presence of B2A-CIC and aPL was evaluated in the pre-transplant serum sample used for crossmatch. Three groups were formed: Group-0: Control subcohort that includes the patients negative for IgA aB2GP1 (*N* = 104). Group-1: patients positive for both: antibodies IgA aB2GP1 and presence of B2A-CIC (*N* = 19). Group-2: Patients positive for IgA aB2GP1 but negative for the presence of B2A-CIC (*N* = 28).

**Figure 1:**
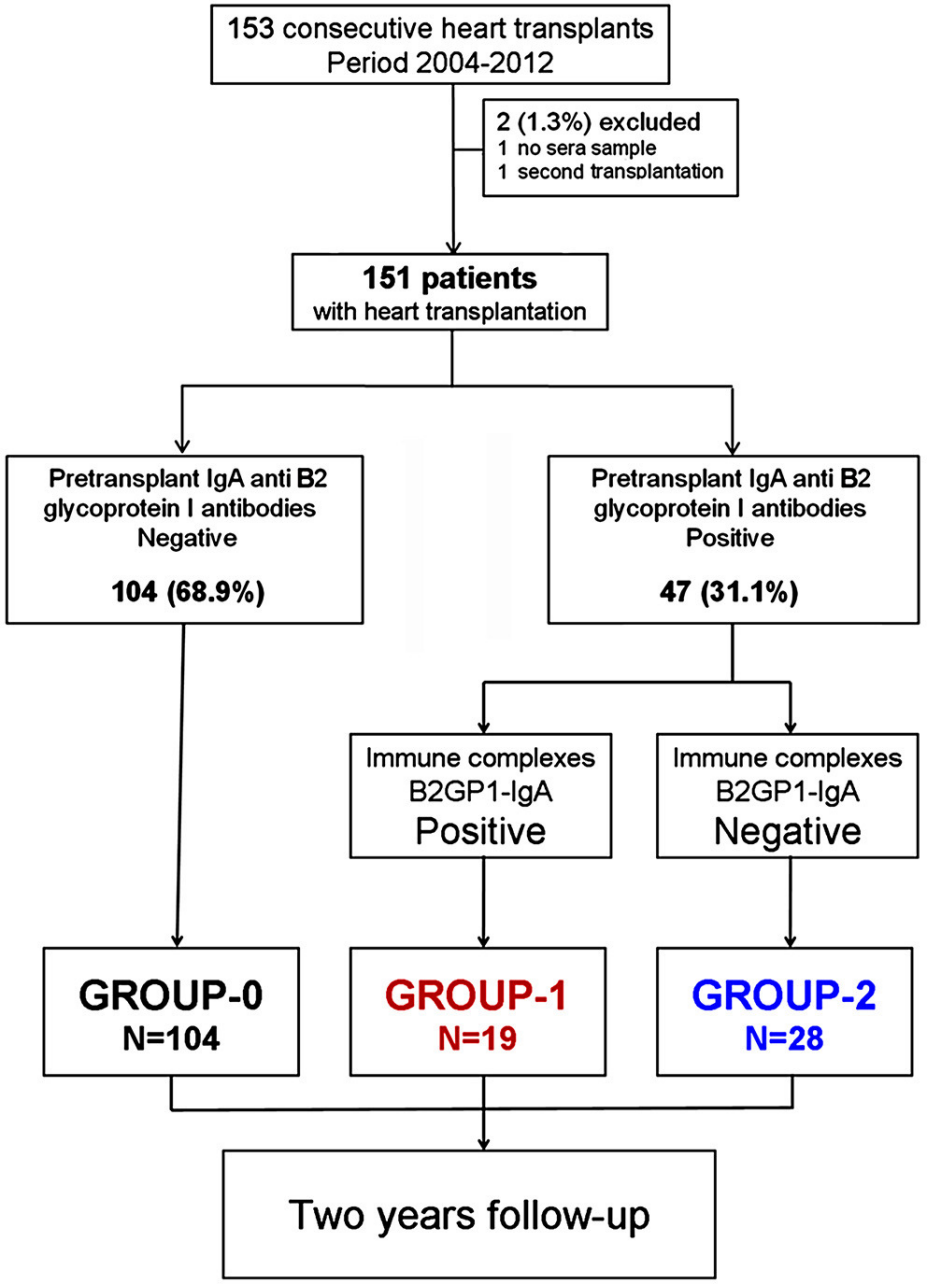
Disposition and groups of study.

The disposition algorithm is described in Figure 1.

### Ethical Issues

The study was submitted to the Institutional Review Board (ECCR) of Hospital 12 de Octubre and received a favorable report (Reference Number CEIC-15/008). Since this was a non-interventional observational study and no genetic material was used, following Spanish regulations, informed consent was not required.

### Database

The recipient database includes pretransplant characteristics, these being age, blood type, body mass index, original disease and other associated diseases, cardiovascular risk factors (arterial hypertension, hyperlipidemia, diabetes, and smoking) and immunological data. Posttransplant characteristics included data related to donors' features, immunosuppressive treatment, incidence of thrombotic and cardiovascular events, enablers factors for thrombotic events, patient survival and causes of mortality.

### Post-operative Immunosuppressive Treatment

This consists in: (1) Two intravenous bolus of basiliximab (20 mg) on days 0 and 4 after transplant. (2) Cyclosporine (CsA), 5– 8 mg/kg per day during the first year (to maintain serum CsA level within the range of 250–350 ng/mL). (3) Mofetil mycophenolate (MMF) 2–3 grams per day. (4) Steroids intravenously, methylprednisolone 500 mg, before and during surgery. After operation, 125 mg every 8 h for 3 doses. This therapy was followed by oral prednisone (1 mg/kg per day, tapered by 0.1 mg/kg on alternate days to 0.2 mg/kg per day and reduced to 0.1 mg/kg per day after 1 year).

### Definitions

Early Graft Failure (EGF): a graft endpoint produced by the death of the patient or a re-transplant associated with graft failure within the first 30 days after transplantation.

Thrombotic events: venous, arterial or intracavitary thrombosis, pulmonary thromboembolism, thrombotic microangiopathy, transient ischemic attack or acute stroke. Events were diagnosed by imaging techniques or histologic study.

Enablers for thrombotic events: mechanical support (IABP, ECMO), atrial arrhythmias, surgery and catheters, infections or sepsis.

Cardiovascular events: myocardial infarction, coronary revascularization, thrombotic events, death or re-transplant.

### Laboratory Determinations

Autoantibodies were measured in pre-transplant serum used for crossmatch or in a serum sample obtained in the month before transplantation. Anti-cardiolipin or anti-B2GPI of IgG and IgM isotypes were evaluated using the BioPLex 2200 multiplex immunoassay system (Biorad, Hercules CA, USA). Antibody levels above 18 U/mL were considered positive (99th percentile of healthy population).

IgA aCL and aBGPI antibodies were quantified by enzyme-linked immunosorbent assays (ELISA) using IgA-aCL and IgA-aB2GPI QUANTA Lite (INOVA Diagnostics Inc., San Diego, CA, USA). Antibody levels above 20 U/mL were considered positive. Cutoff were established with the 99th percentile of a healthy population in our hospital and coincided with the cutoff suggested in the assay manufacturer's guidelines (25).

Mean levels of anticardiolipin antibodies were: IgG 1.9 ± 0.2 and IgM 1.7 ± 0.3 IU/ml. Antibodies anti-B2GPI mean levels were: IgG 2.0 ± 0.4 IU/ml and IgM 1.8 ± 0.4 IU/ml. No significant differences were observed between the percentage of patients who were positive for anti-cardiolipin or anti-B2GPI (IgG/IgM isotypes) vs. healthy people. No association was found between the presence of IgG/IgM aPL and post-transplant mortality.

### Quantification of Immune Complexes B2GP1-IgA

Determination of B2A-CIC levels was performed as previously described (22). Briefly, 96 wells Nunc maxisorp™ plates (A/S Nunc, Kamstrup, Roskilde, Denmark) were coated with mouse monoclonal antibody anti-human B2GP1 H219 (Mabtech AB, Nacka Strand, Sweden) at 2 μg/mL in PBS pH 7.4 and incubated 16 h at 4°C. The coated plates were washed three times (PBS 0.1% tween 20) and blocked 30 min at room temperature with PBS + 1% bovine serum albumin (Sigma-Aldrich, St. Louis, MO, USA) (RT). Blocked plates were washed (PBS 0.1% tween 20, 3X) and patients serum diluted at 1:100 in PBS were dispensed (100 μL/well; duplicates) and incubated 2 h at room temperature. After incubation, plates were washed (PBS 0.1% tween 20, 3X) and Anti-human IgA HRP-conjugate (INOVA) was used to detect B2A-CIC. The concentration of B2A-CIC of each serum was obtained by interpolating the mean optical density values with a calibration curve. Cutoff for B2A-CIC (21UA) was established with the 99th percentile of a healthy population in our hospital. These levels were practically identical to those calculated through the analysis of ROC curves (22). All the procedures were performed in a Triturus^®^ Analyzer (Diagnostics Grifols, S.A. Barcelona, Spain).

### Statistical Methods

Association between qualitative variables was determined with Pearson's Chi-square test or Fisher's exact test, when appropriate. Results were expressed as absolute frequency and percentage. The relative measure of an effect was expressed as odds ratio.

Prior to the comparison of scaled variables, assessment of normality distribution with Kolmogorov-Smirnov test was performed. The Student's *T*-test was used for comparisons of scaled variables that followed a normal distribution and the results were expressed as mean ± standard error. Mann-Whitney U test was used for comparisons when the outcome was not normally distributed. Results were expressed as median and interquartile range.

Patients' survival probability and incidence of events were calculated using the Kaplan-Meier method. The differences between the survival distributions were evaluated with the Log-rank test and the relative measure of a condition on survival was expressed as hazard ratio (HR).

Odds Ratio calculations and Multivariate analysis were performed using logistic regression model. Multivariate analysis of 3-months mortality-associated risk factors was also performed using logistic regression because it is a more effective model than the Cox regression for events concentrated in a short period of time (26). Adjustment of *p*-values for multiple comparisons were obtained by the false discovery rate method (27). Probabilities under 0.05 were considered significant.

Data were processed and analyzed using MedCalc for Windows version 18.9 (MedCalc Software, Ostend, Belgium) and the “R” programming language (R Foundation for Statistical Computing, Vienna, Austria) (28).

## Results

### Pre-transplant Characteristics of the Three Groups

Of the patients positive for IgA aB2GP1, a total of 19 had levels of B2A-CIC above the cutoff point (Group-1), the remaining 28 were negative for the presence of B2A-CIC (Group-2). No significant differences in the pre-transplant characteristics among patients in the three Groups were found (Table 1).

**Table 1:** Pre-transplant characteristics of patients on Group-1, Group-2, and Group-0 and main outcomes after transplant.

	**Group-1** ***N*** **=** **19**		**Group-2** ***N*** **=** **28**		**Group-0** ***N*** **=** **104**		**Significance**		
	***N*/median**	**%/IQR**	***N*/median**	**%/IQR**	***N*/median**	**%/IQR**	**G1 vs. G0**	**G2 vs. G0**	**G1 vs. G2**
Sex (female)	4	(21.1%)	8	(28.6%)	16	(15.4%)	N.S.	N.S.	N.S.
Age	55.0	44.0–64.0	53.5	39.0–58.5	49.5	39.0–58.5	N.S.	N.S.	N.S.
Body mass index	23.3	21.1–28.6	23.6	21.4–26.5	24.6	22.4–26.4	N.S.	N.S.	N.S.
Blood type									
Group 0	8	(42.1%)	12	(42.9%)	48	(46.2%)	N.S.	N.S.	N.S.
Group A	9	(47.4%)	13	(46.4%)	41	(39.4%)	N.S.	N.S.	N.S.
Group B	2	(10.5%)	3	(10.7%)	10	(9.6%)	N.S.	N.S.	N.S.
Group AB	0	(0%)	0	(0%)	5	(4.8%)	N.S.	N.S.	N.S.
Rh positive	18	(94.7%)	25	(89.3%)	85	(81.7%)	N.S.	N.S.	N.S.
Causes of heart dysfunction									
Ischemic	7	(36.8%)	10	(35.7%)	27	(26%)	N.S.	N.S.	N.S.
Idiopathic	6	(31.6%)	7	(25%)	45	(43.3%)	N.S.	N.S.	N.S.
Restrictive	0		4	(14.3%)	5	(4.8%)	N.S.	N.S.	N.S.
Valvular	4	(21.1%)	1	(3.6%)	6	(5.8%)	N.S.	N.S.	N.S.
Others	2	(10.5%)	6	(21.4%)	21	(16.4%)	N.S.	N.S.	N.S.
Pathologies and risk factors									
Renal dysfunction	3	(15.8%)	4	(14.3%)	21	(20.2%)	N.S.	N.S.	N.S.
HTA antecedents	4	(21.1%)	6	(21.4%)	33	(31.7%)	N.S.	N.S.	N.S.
Diabetes	1	(5.3%)	6	(21.4%)	25	(24%)	N.S.	N.S.	N.S.
Dyslipidemia	7	(36.8%)	9	(32.1%)	33	(25.8%)	N.S.	N.S.	N.S.
Hyperuricemia	3	(15.8%)	1	(3.6%)	14	(13.5%)	N.S.	N.S.	N.S.
Hyperbilirubinemia	2	(10.5%)	7	(25%)	25	(24%)	N.S.	N.S.	N.S.
ALT/AST High levels	3	(15.8%)	4	(14.3%)	30	(28.8%)	N.S.	N.S.	N.S.
Active smoker	4	(21.1%)	7	(25%)	24	(23.1%)	N.S.	N.S.	N.S.
Ex-smoker	4	(21.1%)	8	(28.6%)	29	(27.9%)	N.S.	N.S.	N.S.
No-smoking	11	(57.9%)	13	(46.4%)	51	(49%)	N.S.	N.S.	N.S.
Patients with thrombotic antecedents^*^	0		1	(2.1%)	6	(5.8%)	N.S.	N.S.	N.S.
Deep venous thrombosis	0		1	(2.1%)	3	(2.9%)	N.S.	N.S.	N.S.
Pulmonary embolism	0		1	(2.1%)	4	(3.8%)	N.S.	N.S.	N.S.
Previously anticoagulated	13	(68.4%)	15	(53.6%)	56	(53.8%)	N.S.	N.S.	N.S.
Other vascular diseases									
Thrombosis A/V	0		1	(3.6%)	8	(7.7%)	N.S.	N.S.	N.S.
Thrombophebitis	0		0		2	(1.9%)	N.S.	N.S.	N.S.
Ethnicity Caucasian	18	(94.7%)	27	(97.4%)	100	(96.2%)	N.S.	N.S.	N.S.
Ethnicity: others	1	(5.3%)	1	(2.6%)	4	(3.8%)	N.S.	N.S.	N.S.
Post-transplant main outcomes									
Dead in 2 years	8	(42.1%)	7	(25%)	14	(13.5%)	*P* = 0.009	N.S.	N.S.
Dead in 3 months	8	(42.1%)	5	(17.9%)	10	(9.6%)	*P* = 0.006	N.S.	N.S.
Dead from month 4–24	0		2	(9% of 23)	4	(4% of 94)	N.S.	N.S.	N.S.
Patients with thrombotic events^*^	10	(52.6%)	13	(46.4%)	27	(26.0%)	*P* < 0.001	*P* = 0.060	N.S.
Intracavitary thrombus	4	(21.1%)	2	(7.1%)	0	(0%)	*P* < 0.001	N.S.	N.S.
Stroke	3	(15.8%)	2	(7.1%)	3	(2.9%)	N.S.	N.S.	N.S.
Deep venous thrombosis	0	(0%)	0	(0%)	2	(1.9%)	N.S.	N.S.	N.S.
Pulmonary embolism	0	(0%)	1	(3.6%)	1	(1%)	N.S.	N.S.	N.S.
Arterial Thrombosis	1	(5.3%)	1	(3.6%)	1	(1%)	N.S.	N.S.	N.S.
Patients with thrombosis or dead	14	(73.7%)	11	(39.3%)	17	(16.3%)	*P* < 0.001	*P* = 0.013	*P* = 0.020

*IQR, interquartile range; G1, Group 1; G2, Group 2; G0, Group 0; N.S., not significant*.

* *Some patients have more than one event. P-values were adjusted for multiple comparisons*.

A higher incidence of thrombocytopenia was observed in groups 1 and 2 compared to Group-0. Although the differences were initially significant, the significance was lost (not shown) after performing the adjustment for multiple comparisons.

### Post-transplant Survival Evolution

**Figure 2:**
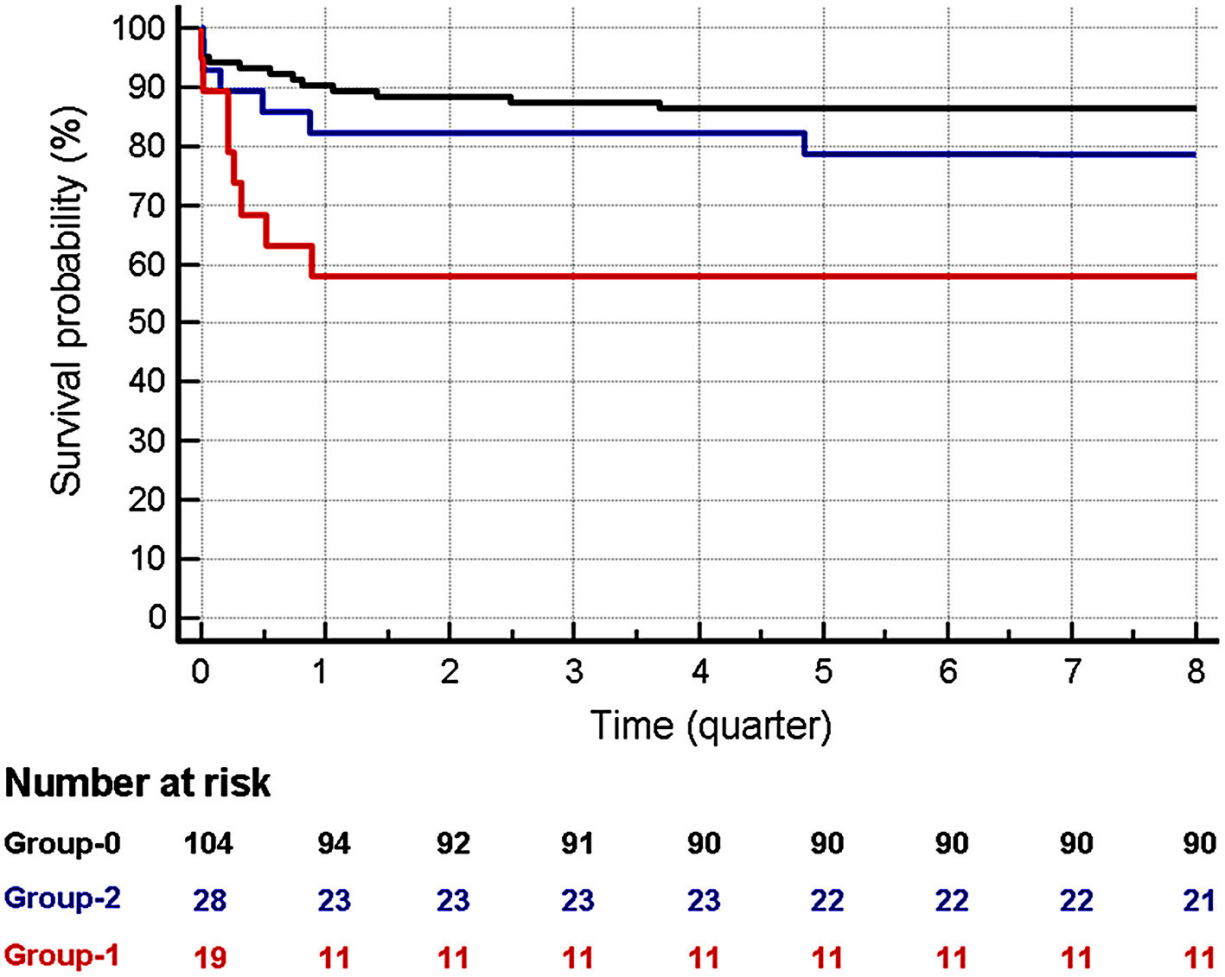
Survival at 2 years in patients in the three groups. Red: Group-1. Blue: Group-2. Dark: Group-0. The time is indicated in quarters. Mortality in group-1 is higher than in group-0, both at 3 months (HR:5.08; 95% CI: 1.36–19.01) and at 2 years (HR: 3.82; 95% CI: 1.15–12.66). No significant differences were observed between patients in Group-2 vs. Group-0 both at 3 and 24 months.

When the survival of the three groups was compared at 2 years after transplantation, a significantly higher mortality was found in Group-1 vs. Group-0 patients. This mortality was especially concentrated in the first trimester (Figure 2). Non-significant differences were observed in the causes of death among the 3 groups (not shown). The Kaplan Meier 2-year survival analysis for the patients in Group-1 vs. Group-0 showed a Hazard ratio (HR) of 3.82 (95% CI: 1.54–12.66). No significant differences were observed between the patients in Group-2 vs. Group-0 (HR: 1.93; 95% CI: 0.75–5.01) (Figure 2 and Table 1).

When we performed the Kaplan Meier survival analysis focusing on the first 3 months after transplantation, the mortality in Group-1 vs. Group-0 was still higher than that observed at 2 years: HR 5.08 (95% CI: 1.36–19.01). Mortality of patients in Group-1 was higher than in Group-2 (HR: 2.65) but these differences were not significant (HR 95% CI: 0.56–12.54). After the first trimester (from the fourth month to the end of the second year), no significant differences were observed in the survival among the three groups (Table 1).

When we compared the differences between deceased patients in the first trimester vs. those who survived (Table 2), we found that there was a significantly higher proportion of women, blood type group A and positivity of B2A-CIC in the dead patients. No significant differences were observed in the rest of the characteristics, including the proportion of patients who were positive for IgA aB2GP1 but negative for B2A-CIC.

**Table 2:** **(A)** Pre-transplant characteristics of patients who died in the first trimester after heart transplantation vs. alive patients. **(B)** Logistic regression multivariate analysis of factors associated with mortality in the first trimester after heart transplantation.

**(A) Pre-transplant characteristics**	**Dead** ***N*** **=** **23**		**Alive** ***N*** **=** **128**		***P*-value**
**Variable**	***N*/median**	**%/IQR**	***N*/median**	**%/IQR**	
Sex (female)	8	(34.8%)	20	(15.6%)	0.030
Age	52	42.3–61.0	51	39.0–58.5	N.S.
Body mass index	23.7	20.7–27.9	24.5	22.0–26.6	N.S.
IgA aB2GP1 positive and B2A-CIC positive	8	(34.8%)	11	(8.6%)	<0.001
IgA aB2GP1 positive and B2A-CIC negative	5	(21.7%)	23	(18%)	N.S.
Blood type					
Group 0	7	(30.4%)	61	(47.7%)	N.S.
Group A	15	(65.2%)	48	(37.5%)	0.013
Group B	1	(4.3%)	14	(10.9%)	N.S.
Group AB	0	(0%)	5	(3.9%)	N.S.
Rh positive	21	(91.3%)	107	(83.6%)	N.S.
Other pathologies and risk factors					
Renal dysfunction	5	(21.7%)	23	(18%)	N.S.
HTA antecedents	5	(21.7%)	38	(29.7%)	N.S.
Diabetes	3	(13%)	29	(22.7%)	N.S.
Dyslipidemia	8	(34.8%)	41	(32%)	N.S.
Hyperuricemia	1	(4.3%)	17	(13.3%)	N.S.
Hyperbilirubinemia	9	(39.1%)	25	(19.5%)	N.S.
ALT/AST High levels	3	(13%)	34	(26.6%)	N.S.
Active smoker	7	(30.4%)	28	(21.9%)	N.S.
Ex-smoker	5	(21.7%)	36	(28.1%)	N.S.
No-smoking	11	(47.8%)	64	(50%)	N.S.
Patients with thrombotic antecedents	1	(4.3%)	6	(5.8%)	N.S.
Previously anticoagulated	15	(65.2%)	69	(53.9%)	N.S.
Other vascular diseases					
Thrombosis A/V	1	(4.3%)	8	(6.3%)	N.S.
Thrombophlebitis	0	(0%)	2	(1.6%)	N.S.
Ethnicity Caucasian	22	(95.7%)	123	(96.1%)	N.S.
Ethnicity: others	1	(4.3%)	5	(3.9%)	N.S.
**(B) Multivariate analysis**	**Univariate**		**Multivariate**		
**Variable**	**OR**	**95% CI OR**	**OR**	**95% CI OR**	***P*****-value**
B2A-CIC positive	5.67	1.97–16.33	6.13	1.93–19.40	0.002
Sex (female)	2.88	1.08–7.67	4.18	1.35–12.94	0.013
Blood type: Group A	3.13	1.23–7.92	4.05	1.44–11.43	0.008

*IQR, interquartile range; N.S., not significant*.

When these factors associated with mortality were subjected to logistic regression multivariate analysis, it was found that the presence of B2A-CIC (OR: 6.13; 95% CI: 1.93–19.40), blood type A (OR: 4.05; 95% CI: 1.44–11.43) and female gender (OR: 4.18; 95% CI: 1.35–12.94) were independently associated with early mortality (Table 2).

### Post-transplant Thrombotic Events

Thrombotic events were only observed in the first trimester after transplantation. After this period, no event consistent with the APS clinical classification criteria were observed. The Group-1 had a significantly higher incidence of thrombotic events than Group-0 (OR: 9.53; 95% CI: 2.75–33.07). No significant differences were observed in patients in Group-2 vs. Group-0 or Group-1 (Table 1). The post-transplant thrombosis observed most was intracavitary Cardiac thrombus with a significantly higher incidence in Group-1 vs. Group-0 (OR: 60.7; 95% CI: 3.12–1182).

Patients who suffered thrombotic events in the first 3 months compared with patients who did not suffer these events (Table 3) had a significantly higher proportion of B2A-CIC positive patients (OR: 6.42; 95% CI: 2.10–19.63) and pre-transplant active smokers (OR: 3.14; 95% CI: 1.13–8.72). No significant differences were observed in the remaining clinical characteristics (Table 3). In the multivariate analysis, it was confirmed that the pre-transplant presence of B2A-CIC (OR: 7.52; 95% CI: 2.30–24.56) and the habit of smoking actively (OR: 3.78; 95% CI: 1.25–11.41) were independently associated with incident of thrombotic events in the first trimester alter heart transplantation (Table 3).

**Table 3:** **(A)** Pre-transplant characteristics of patients who suffer thrombotic events in the first trimester after heart transplant vs. those without these events. **(B)** Logistic regression multivariate analysis of pre-transplant factors associated with incidence of thrombotic events in the first 3 months after heart transplantation.

**(A) Pre-transplant characteristics**	**Patients with events** ***N*** **=** **18**		**Patients without events** ***N*** **=** **133**		***P*-value**
**Variable**	***N*/median**	**%/IQR**	***N*/median**	**%/IQR**	
Sex (female)	3	(16.7%)	25	(18.8%)	N.S.
Age	50.5	40.0–58.0	52	39.0–59.0	N.S.
Body mass index	25.5	20.9–29.2	24.4	22.1–26.4	N.S.
**IgA aB2GP1** **+** **and B2A-CIC positive**	7	(38.9%)	12	(9%)	<0.001
IgA aB2GP1 + and B2A-CIC negative	5	(27.8%)	23	(17.3%)	N.S.
Blood type					
Group 0	9	(50%)	59	(44.4%)	N.S.
Group A	7	(38.9%)	56	(42.1%)	N.S.
Group B	1	(5.6%)	14	(10.5%)	N.S.
Group AB	1	(5.6%)	4	(3%)	N.S.
Rh positive	16	(88.9%)	112	(84.2%)	N.S.
Pathologies and risk factors					
Renal dysfunction	2	(11.1%)	26	(19.5%)	N.S.
HTA antecedents	5	(27.8%)	38	(28.6%)	N.S.
Diabetes	2	(11.1%)	30	(22.6%)	N.S.
Dyslipidemia	7	(38.9%)	42	(31.6%)	N.S.
Hyperuricemia	1	(5.6%)	17	(12.8%)	N.S.
Hyperbilirubinemia	1	(5.6%)	33	(24.8%)	N.S.
ALT/AST High levels	7	(38.9%)	30	(22.6%)	N.S.
Active smoker	8	(44.4%)	27	(20.3%)	0.023
Ex-smoker	4	(22.2%)	37	(27.8%)	N.S.
No-smoking	6	(33.3%)	69	(51.9%)	N.S.
Patients with thrombotic antecedents	1	(5.6%)	6	(4.5%)	N.S.
Previously anticoagulated	9	(50%)	75	(56.4%)	N.S.
Other vascular diseases					
Thrombosis A/V	2	(11.1%)	7	(5.3%)	N.S.
Thrombophlebitis	0		2	(1.5%)	N.S.
Ethnicity Caucasian	16	(88.9%)	129	(97%)	N.S.
Ethnicity: others	2	(11.1%)	4	(3%)	N.S.
**(B) Multivariate analysis**	**Univariate**		**Multivariate**		
**Variable**	**OR**	**95% CI OR**	**OR**	**95% CI OR**	***P*****-value**
B2A-CIC positive	6.42	2.10–19.63	6.13	1.93–19.40	0.002
Active smoker	3.14	1.13–8.72	4.18	1.35–12.94	0.013

*IQR, interquartile range; N.S., not significant*.

### Patients Suffering Thrombosis or Death

If we consider grouping early outcomes (thrombosis and death in the first 3 months) as a new variable: thrombosis or death (TRB-D), 73.7% of patients in Group-1 suffer from TRB-D. This proportion is significantly higher than the 16.3% observed in Group-0 (OR: 15.40; 95% CI: 4.87–48.72) and that 39.3% of Group-2 (OR: 5.04; 95% CI: 14.0–18.14) (Table 1).

**Figure 3:**
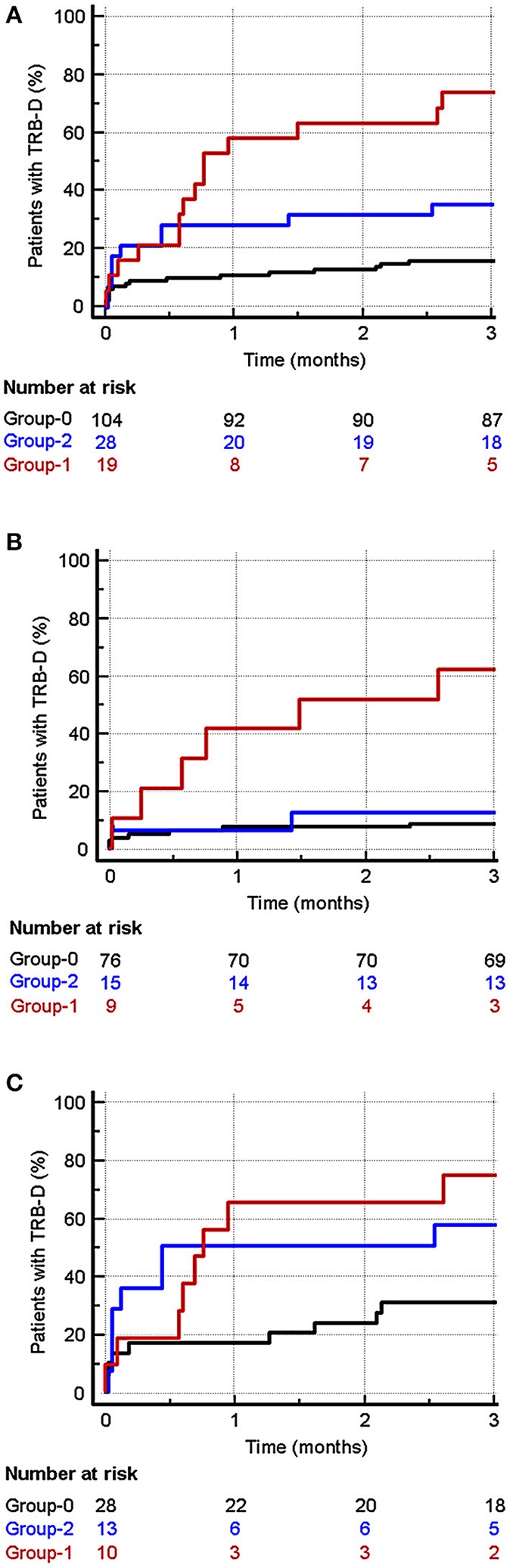
Incidence of TRB-D (thrombotic events or death) in the first 3 months after heart transplantation. Red: Group-1. Blue: Group-2. Dark: Group-0. **(A)** Considering all the patients, the incidence was significantly higher in Group-1 vs. Group-0 (HR: 6.29; 95% CI: 2.22–12.79) and also in Group-2 vs. Group-0 (HR: 2.64; 95% CI: 1.15–6.07). **(B)** In patients without post-transplant risk factors for thrombosis, the incidence of TRB-D in the first trimester was significantly higher in Group-1 patients (B2A-CIC positive) vs. patients of Group-0 (HR 9.03; 95% CI: 1.28–63.65). No significant differences were observed between patients in Group-2 and those in Group-0 (HR: 1.46; 95% CI: 0.35–6.05). **(C)** In patients with risk factors for thrombosis, a significant higher incidence of TRB-D in Group-1 vs. Group-0 (HR: 3.27; 95% CI: 1.11–9.64) can also be observed. No significant differences were found between Group-2 vs. Group-0 (HR: 2.43; 95% CI: 0.92–6.43) and between Group-1 and Group-2 (HR: 1.30; 95% CI: 0.37–4.59).

The Kaplan Meier survival analysis (Figure 3A) confirms that the incidence of TRB-D was significantly higher in Group-1 vs. Group-0 (HR: 6.29; 95% CI: 2.22–12.79) and also in Group-2 vs. Group-0 (HR: 2.64; 95% CI: 1.15–6.07).

Evaluating the characteristics of the 40 patients who suffered TRB-D during the first trimester post-transplant and comparing them with the 111 who remained alive and without events, we found a significantly higher presence of positive B2A-CIC (OR: 11.42; 95% CI: 3.77–34.55), pre-transplant active smokers (OR: 2.09; 95% CI: 1.00–4.34) and blood type A (OR: 2.31; 95% CI: 1.03–5.16) (Table 4).

**Table 4:** **(A)** Pre-transplant characteristics of patients who suffer both, thrombotic events or death (TRB-D) in the first trimester after heart transplant vs. patients who are alive and without thrombotic events at the end of this period. **(B)** Logistic regression multivariate analysis of factors associated with incidence of TRB-D in the first 3 months after heart transplantation.

**(A) Pre-transplant characteristics**	**Death or events** ***N*** **=** **40**		**Live and without events** ***N*** **=** **111**		***P*-value**
**Variable**	***N*/median**	**%/IQR**	***N*/median**	**%/IQR**	
Sex (female)	10	(25%)	18	(16.2%)	N.S.
Age	51.5	40–61	50.0	39–58.8	N.S.
Body mass index	23.9	20.8–28.1	24.5	22.3–26.4	N.S.
**IgA aB2GP1** **+** **and B2A-CIC positive**	14	(35%)	5	(4.5%)	<0.001
IgA aB2GP1 + and B2A-CIC negative	10	(25%)	18	(16.2%)	N.S.
Blood type					
Group 0	15	(37.5%)	53	(47.7%)	N.S.
Group A	22	(55%)	41	(36.9%)	N.S.
Group B	2	(5%)	13	(11.7%)	N.S.
Group AB	1	(2.5%)	4	(3.6%)	N.S.
Rh positive	37	(92.5%)	91	(82%)	N.S.
Pathologies and risk factors					
Renal dysfunction	7	(17.5%)	21	(18.9%)	N.S.
HTA antecedents	10	(25%)	33	(29.7%)	N.S.
Diabetes	5	(12.5%)	27	(24.3%)	N.S.
Dyslipidemia	15	(37.5%)	30	(30.6%)	N.S.
Hyperuricemia	2	(5%)	16	(14.4%)	N.S.
Hyperbilirubinemia	10	(25%)	24	(21.6%)	N.S.
ALT/AST High levels	10	(25%)	27	(24.3%)	N.S.
Active smoker	14	(35%)	21	(18.9%)	N.S.
Ex-smoker	9	(22.5%)	32	(28.8%)	N.S.
No-smoking	17	(42.5%)	58	(52.3%)	N.S.
Patients with thrombotic antecedents	2	(5.0%)	5	(4.5%)	N.S.
Pre transplant prophylactic anticoagulation	23	(57.5%)	61	(55%)	N.S.
Other vascular diseases					
Thrombosis A/V	3	(7.5%)	6	(5.4%)	N.S.
Thrombophlebitis	0		2	(1.8%)	N.S.
Ethnicity Caucasian	37	(92.5%)	108	(97.3%)	N.S.
Ethnicity: others	3	(7.5%)	3	(2.7%)	N.S.
**(B) Multivariate analysis**	**Univariate**		**Multivariate**		
**Variable**	**OR**	**95% CI OR**	**OR**	**95% CI OR**	***P*****-value**
IgA aB2GP1 positive and B2A-CIC positive	11.42	3.77–34.55	13.25	4.14–42.36	<0.001
Blood type: Group A	2.09	1.00–4.34.	2.27	0.98–5.26	0.056
Active smoker	2.31	1.03–5.16	2.58	1.05–6.31	0.038
Sex (female)	0.72	0.72–4.13	1.99	0.72–5.52	0.185

*IQR, interquartile range; N.S., not significant*.

A multivariate analysis was carried out with the three factors associated significantly with TRB-D in the univariate analysis and we also added gender. Although gender did not show an association with TRB-D in the univariate analysis, it was included because gender was previously independently associated with early death.

The pre-transplant habit of smoking actively (OR: 3.92; 95% CI: 1.29–11.9) and the presence of B2A-CIC (OR: 7.75; 95% CI: 2.35–25.61) were the only independent risk factors for the appearance of death or thrombotic events in the first trimester after transplantation (Table 5).

**Table 5:** Logistic regression multivariate analysis (*p* < 0.0001) of pre-transplant and post-transplant predisposing factors associated to TRB-D (thrombosis or death) in the first 3 months after heart transplantation.

	**Univariate analysis**			**Multivariate analysis**		
**Variable**	**OR**	**95% CI OR**	***P*-value**	**OR**	**95% CI OR**	***P*-value**
IgA B2GP1 positive and B2A-CIC positive	11.42	3.77–34.55	<0.001	13.13	3.80–45.36	<0.001
Blood type: Group A	2.09	1.00–4.34.	0.049	1.98	0.83–4.73	0.122
Pre-transplant active smoker	2.31	1.03–5.16	0.042	2.65	1.01–6.94	0.047
Post-transplant predisposing factors	5.45	2.51–11.84	<0.001	5.26	2.19–12.64	<0.001

### Influence of Post-transplant Risk Factors for Thrombotic Events

After transplantation, a total of 50 patients (33%) showed risk factors for the appearance of thrombotic events that were not present before the transplant. The proportion of patients with these factors was somewhat higher in patients in groups 1 and 2 compared with Group-0 (Supplementary Figure 1), although these differences were not found to be significant after a statistical study of multiple comparisons was conducted (Supplementary Table 1).

Kaplan Meier survival analysis of the incidence of TRB-D in patients who were not exposed to risk factors (Figure 3B) showed that Group-1 patients (B2A-CIC positive) have a significantly higher risk of developing TRB-D events in the first trimester vs. patients of Group-0 (HR 9.03; 95% CI: 1.28–63.65). No significant differences were observed between patients in Group-2 and those in Group-0 (HR: 1.46; 95% CI: 0.35–6.05).

In the Kaplan Meier survival analysis of patients with risk factors (Figure 3C), the differences of incidence of TRB-D in Group-1 vs. Group-O group were significant (HR: 3.27; 95% CI: 1.11–9.64) but lower than in the patients without additional risk factor. No significant differences were found between Group-2 vs. Group-0 (HR: 2.43; 95% CI: 0.92–6.43) and between Group-1 and Group-2 (HR: 1.30; 95% CI: 0.37–4.59).

The multivariate analysis including pre-transplant and post-transplant risk factors associated significantly with the occurrence of TRB-D events in the univariate analysis (Table 5) demonstrated that the presence of B2A-CIC is the main independent risk factor for the appearance of TRB-D in the three 3 months after cardiac transplantation (OR: 13.13; 95% CI: 3.80–45.36).

The presence of post-transplant risk factors (OR: 5.26; 95% CI: 2.19–12.64) and the pre-transplant smoking habit (OR: 2.65; 95% CI: 1.01–6.94) also behaved as independent risk factors for TRB-D. The area under the ROC curve of this multivariate analysis was 0.818 (95% CI: 0.747–0.876).

## Discussion

In this work, for the first time we have described that the pre-transplant presence of B2A-CIC identifies a subgroup of patients prone to develop the worst outcomes after heart transplant: death and thrombotic events.

The presence of aPL is not enough to trigger a thrombotic event. Additional factors that involve the activation of the mechanisms of innate immunity in the context of processes such as infection or surgery need to converge. It is what is known as the hypothesis of the two hits to provoke the event: the presence of aPL would be a “first hit” and the concurrent factors would be the “second hit” (29).

The follow-up of patients who are carriers of antiphospholipid antibodies (first hit) and who will undergo transplant surgery (second hit) is an excellent tool to study primary APS because it allows us to have extensive clinical information and serum samples from the stage prior to the triggering of the thrombotic event.

Most patients with primary APS are studied after the event appears. The high prevalence of aPL (first “hit”) in patients with end-stage renal disease or severe heart failure, together with their exposure to transplant surgery (a well-known “second hit”), triggers that a high percentage of these patients will have thrombotic events after transplantation. To perform a similar study with patients of the general population, where the prevalence of aPL is ~2% and the incidence of events/year in aPL carriers is 3% (30–33), it would be necessary to follow-up ~40,000 individuals for 1 year.

### Pre-transplant Markers and Mortality

The presence of B2A-CIC allows us to identify which IgA aB2GP1 patients have the highest mortality and that the survival in patients B2A-CIC negative does not differ from that observed in the control group. Other independent risk factors for early mortality after heart transplantation are the sex (women) and the positivity of the blood group A.

Female gender is a known risk factor for early mortality in heart transplant patients: in the 23rd official adult heart transplantation report (2006) of the International Registry of Heart Transplantation for adult patients, female gender was associated with higher mortality (34). Although this fact has been attributed to a higher incidence of acute graft failure, the mechanisms responsible for these gender-based differences have yet to be understood.

The presence of group A as a risk factor is a different way of seeing the real fact: non-O blood group patients are at more risk for thrombotics events (35). The greatest risk for patients in groups AB and B could not be demonstrated due to the small sample size. The group of patients have a lower risk of developing thrombotic events which has been attributed to have lower factor VIII and von Willebrand factor blood levels. (36, 37).

### Patients With Thrombotics Events

In this work, we have shown that patients who are positive for IgA aB2GP1 and also present B2A-CIC are those having the highest risk of thrombotic events when they are subjected to a situation capable of triggering the occurrence of thrombotic events such as transplant surgery. The risk of those who are negative for B2A-CIC is only a little higher than that found in the control group.

In the first weeks after kidney transplantation, the most serious thrombotic complication is graft thrombosis, which involves graft loss in the vast majority of patients. In the case of heart transplantation, intracavitary thrombosis, or thrombosis in large vessels can be identified and treated, however multiple thrombosis of small vessels are less evident and can lead to organ failure. Since the heart is a unique organ essential for life, the graft loss necessarily implies the death of the patient. Thus, as in kidney transplantation, the presence of B2A-CIC in the heart transplant is also strongly associated with the graft loss, graft loss being considered the death of patients in the first 3 months after transplantation. This work confirms and validates the described previously conclusion with kidney transplant patients that the presence of B2A-CIC in patients positive for IgA aB2GPI is associated with thrombotic risk. It also confirms the observation that patients positive for IgA aB2GPI who are negative for B2A-CIChave the same risk of developing APS events as patients who are negative for anti-B2GP1 antibodies (24).

Mortality and development of thrombotic events after transplantation are multifactorial processes involving several risk factors present mainly before transplantation and also others that arise after transplantation (such as multiple surgery, mechanical support and infections). The post-transplant evolution in patients receiving a heart transplant is more complex than in those who undergo kidney transplantation. The failures in graft function in renal transplantation that may arise in the first post-transplant moments can be easily treated with renal function replacement methods (dialysis) that involves a low degree of intervention. However, the appearance of complications with functional repercussion in the heart transplant as well as the use of invasive methodologies (surgery, catheterization, mechanical ventilation, impulse balloons, etc.) to control these situations have repercussions in all the circulatory tree that favor the appearance of thrombotic events. These situations behave as enhancers of thrombotic activity (post-transplant risk factors).

The incidence of TRB-D events in the first trimester in patients without these post-transplant risk factors is very similar to that observed in the first trimester in renal transplants: B2A-CIC negative barely distinguish themselves from the control group (24). However, in patients where these factors are present, although the incidence of TRB-D events in the B2A-CIC positive is still significantly higher, the incidence of TRB-B triples in group-0 and quadruples in group-2.

This clear lower influence of the enhancers in patients with B2A-CIC is due to the fact that most of these patients are already in a pro-thrombotic state and, therefore, the population susceptible to being influenced by these factors is clearly much lower.

### Predictive Value of APL

The predictive value of the aPL presence for occurrence of APS events is low: only a small proportion of patients positive for aPL included in the classification criteria develop thrombotic events (38). The determination of B2A-CIC in carriers of IgA aB2GP1 makes it possible to discard those with a similar risk to the general population (CIC negative) and to focus attention on those with greater risk (B2A-CIC positive) as well as to establish preventive treatments depending on the remaining clinical characteristics.

The value of extra-criteria aPL is on the rise. Its study has allowed us to recognize that there is no simple mechanism by which obstetric complications are triggered in the pathogenesis of PHC but rather diverse pathologic pathways (TLR, NADPH-ox, LRP8) are being linked to aPL (39, 40). In this way, a new pathophysiological pathway of APL is studied through the presence of immune complexes of B2GP1 and aPL (IgG, IgM, or IgA isotype) and its association with acute events (23) and with extra-criteria manifestations of APS (41).

The pathogenic mechanisms and the biological significance of the presence of CIC are not known. In the majority of patients with antiphospholipid antibodies there is a contradictory situation from the point of view of classical immunology: B2GP1 (the antigen) is a relatively abundant blood protein that circulates and shares space and time with the antibody that recognizes it.

Although protein and antibody are present simultaneously in the blood, they paradoxically do not interact or produce pathological situations in most patients, this only occurring in a minority.

Our group initially proposed a possible explanation as to why there are patients with immunocomplexes and high risk of thrombosis and patients without immune complexes and at low risk the existence of aPL with different affinities for B2GP1 in each patient. Patients with high affinity antibodies would be those who are able to form CIC. The presence of CIC would indicate that the aPL are of high affinity and therefore more pathogenic (22). However, this hypothesis does not explain the observation that the presence of CICs vary throughout the evolution of the disease while the antibody titers remain stable (23).

Based on the work of Agar et al. who describe that anti-B2GP1antibodies can bind to fish-hook shape of B2GP1 but do not bind to the circular shape (42), we propose that the variation in affinity would depend on the antigen (B2GP1). The variations in the antigen affinity for the antibody would be conditioned by the exposure of cryptic epitopes that would not be accessible under physiological conditions (“O” Shape of B2GP1). The epitopes would be accessible after changes in the tertiary structure after the “activation” of the B2GP1 molecule in the context of a defensive response, however the validity of this hypothesis must be tested in subsequent studies.

This study has several limitations notwithstanding the experience of 8 years of consecutive heart transplants. It is a single-center study and the number of patients in small. Therefore, multicenter studies with a higher number of patients are mandatory to confirm these findings and to establish the most appropriate therapeutic approach for these patients. At present and based on our knowledge up to now, we could consider that a prophylactic heparin treatment and an exhaustive vigilance in the B2A-CIC positive patients during the first 3 months after transplantation could help to ameliorate the mortality and thrombotic complications.

In summary, the main finding of this study is that 40% of patients with IgA aB2GP1 also present B2A-CIC. Approximately 74% of B2A-CIC suffer thrombotic events or death after heart transplant, so the positivity of this biomarker identifies patients with higher risk of mortality and incidence of thrombotic events after heart transplantation. There were no significant differences in mortality and incidence of thrombosis between patients who do not have B2A-CIC and those who are negative for aPL of class IgA.

## Data Availability Statement

All datasets generated for this study are included in the article/Supplementary Material.

## Ethics Statement

This study was submitted to the Institutional Review Board (ECCR) of Hospital 12 de Octubre and received a favorable report (Reference Number CEIC-15/008). Since this was a non-interventional observational study and no genetic material was used, following Spanish regulations, informed consent was not required.

## Author Contributions

AS and MS conceived the project, designed the research, and wrote the manuscript. LM and JD were responsible for the patients' care and clinical data collection. MS and JM-F performed the determinations of antiphospholipid antibodies and quantification of immune complexes. EM, DP, and OC-M processed the data from the transplant waiting list, integrated the information with the clinical history, and reviewed the integrity of all the data. MS and LM were responsible for the database. AS and MS carry out the statistical analysis. All authors contributed to the data interpretation, discussed the results, reviewed the manuscript, and agreed with the final version.

### Conflict of Interest

The authors declare that the research was conducted in the absence of any commercial or financial relationships that could be construed as a potential conflict of interest. The reviewer GN declared a past co-authorship with one of the authors AS to the handling editor.

## Supplementary Material

Supplementary Figure 1 Incidence of TRB-D (thrombosis or death) in the 3 first months after transplantation in function of post-transplant risk factors (enhancer for thrombotic activity). Gray: patients with enhancer factors. White: patients without enhancer factors.

## Abbreviations

OR: Odds ratio
HR: Hazards ratio
CI: Confidence interval
aB2GPI: Anti-Beta-2 glycoprotein-I antibodies
aCL: Anti-cardiolipin antibodies
CIC: Circulating immune complexes
B2A-CIC: Circulating immune-complexes of IgA bounded to Beta-2 glycoprotein-I
TRB-D: Presence of a severe post-transplant output defined by thrombosis or death of the patient
IABP: Intra-Aortic Balloon Pump
ECMO: ExtraCorporeal Membrane Oxygenation.
