# Supplementary material for: Immune Complexes of Beta-2-Glycoprotein I and IgA Antiphospholipid Antibodies Identify Patients With Elevated Risk of Thrombosis and Early Mortality After Heart Transplantation
Source: Front Immunol. 2019 Dec 23;10:2891. doi: 10.3389/fimmu.2019.02891 (PMC6935976; doi:10.3389/fimmu.2019.02891)
Supplement: Supplementary file 1 [file Table_1.DOC]

**Supplementary Table 1.** Post-transplant predisposing factors for thrombotic activity in the three groups. * Some patients have more than one event. G1: Group 1. G2: Group 2. G0: Group 0. N.S.: Not significant. P values were adjusted for multiple comparisons. Values <0.05 were considered significant.

|  | Group-1 N=19 | | Group-2 N=28 | | Group-0 N=104 | | Significance | | |
| --- | --- | --- | --- | --- | --- | --- | --- | --- | --- |
|  |  |  |  |  |  |  | G1 vs G0 | G2 vs G0 | G1 vs G2 |
| Patients with predisposing factors* | 10 | (52.6%) | 13 | (46.4%) | 28 | (26.9%) | P=0.073 | P=0.073 | N.S. |
| Atrial arrhythmia | 0 | (0%) | 3 | (10.7%) | 4 | (3.8%) | N.S. | N.S. | N.S. |
| Surgery | 7 | (36.8%) | 6 | (21.4%) | 16 | (15.4%) | N.S. | N.S. | N.S. |
| Central catheter | 0 | (0%) | 0 | (0%) | 3 | (2.9%) | N.S. | N.S. | - |
| Extracorporeal oxygenation | 1 | (5.3%) | 1 | (3.6%) | 1 | (1%) | N.S. | N.S. | N.S. |
| Intra-Aortic Balloon Pump | 0 | (0%) | 1 | (3.6%) | 0 | (0%) | - | N.S. | N.S. |
| Embolectomy | 1 | (5.3%) | 0 | (0%) | 0 | (0%) | N.S. | - | N.S. |
| Infections or sepsis | 0 | (0%) | 1 | (3.6%) | 0 | (0%) | - | N.S. | N.S. |
| Thrombophilia | 0 | (0%) | 2 | (7.1%) | 3 | (2.9%) | N.S. | N.S. | N.S. |
| Others | 2 | (10.5%) | 1 | (3.6%) | 4 | (3.8%) | N.S. | N.S. | N.S. |
