# Supplementary figures and images for: Immune Complexes of Beta-2-Glycoprotein I and IgA Antiphospholipid Antibodies Identify Patients With Elevated Risk of Thrombosis and Early Mortality After Heart Transplantation
Source: Front Immunol. 2019 Dec 23;10:2891. doi: 10.3389/fimmu.2019.02891 (PMC6935976; doi:10.3389/fimmu.2019.02891)

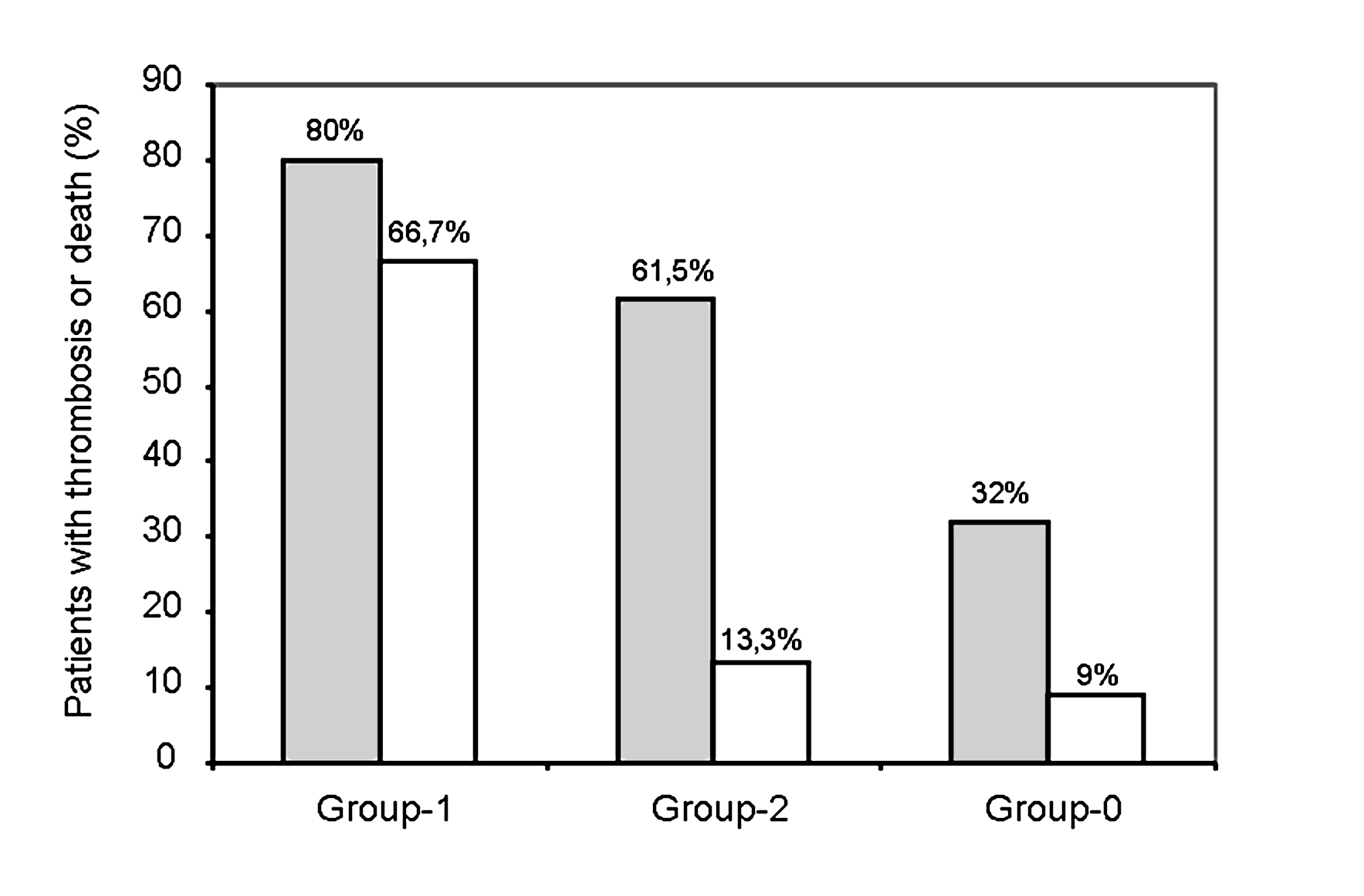

Supplement: Supplementary Figure 1 — Incidence of TRB-D (thrombosis or death) in the 3 first months after transplantation in function of post-transplant risk factors (enhancer for thrombotic activity). Gray: patients with enhancer factors. White: patients without enhancer factors. [file Image_1.TIF]
